# Engineered Cytokine Signaling to Improve CAR T Cell Effector Function

**DOI:** 10.3389/fimmu.2021.684642

**Published:** 2021-06-04

**Authors:** Matthew Bell, Stephen Gottschalk

**Affiliations:** ^1^Department of Bone Marrow Transplantation and Cellular Therapy, St. Jude Children’s Research Hospital, Memphis, TN, United States; ^2^Graduate School of Biomedical Sciences, St. Jude Children’s Research Hospital, Memphis, TN, United States

**Keywords:** CAR T cells, immunotherapy, T Cell therapy, cytokine receptors, cancer, cytokines

## Abstract

Adoptive immunotherapy with T cells genetically modified to express chimeric antigen receptors (CARs) is a promising approach to improve outcomes for cancer patients. While CAR T cell therapy is effective for hematological malignancies, there is a need to improve the efficacy of this therapeutic approach for patients with solid tumors and brain tumors. At present, several approaches are being pursued to improve the antitumor activity of CAR T cells including i) targeting multiple antigens, ii) improving T cell expansion/persistence, iii) enhancing homing to tumor sites, and iv) rendering CAR T cells resistant to the immunosuppressive tumor microenvironment (TME). Augmenting signal 3 of T cell activation by transgenic expression of cytokines or engineered cytokine receptors has emerged as a promising strategy since it not only improves CAR T cell expansion/persistence but also their ability to function in the immunosuppressive TME. In this review, we will provide an overview of cytokine biology and highlight genetic approaches that are actively being pursued to augment cytokine signaling in CAR T cells.

## Introduction

**Figure 1:**
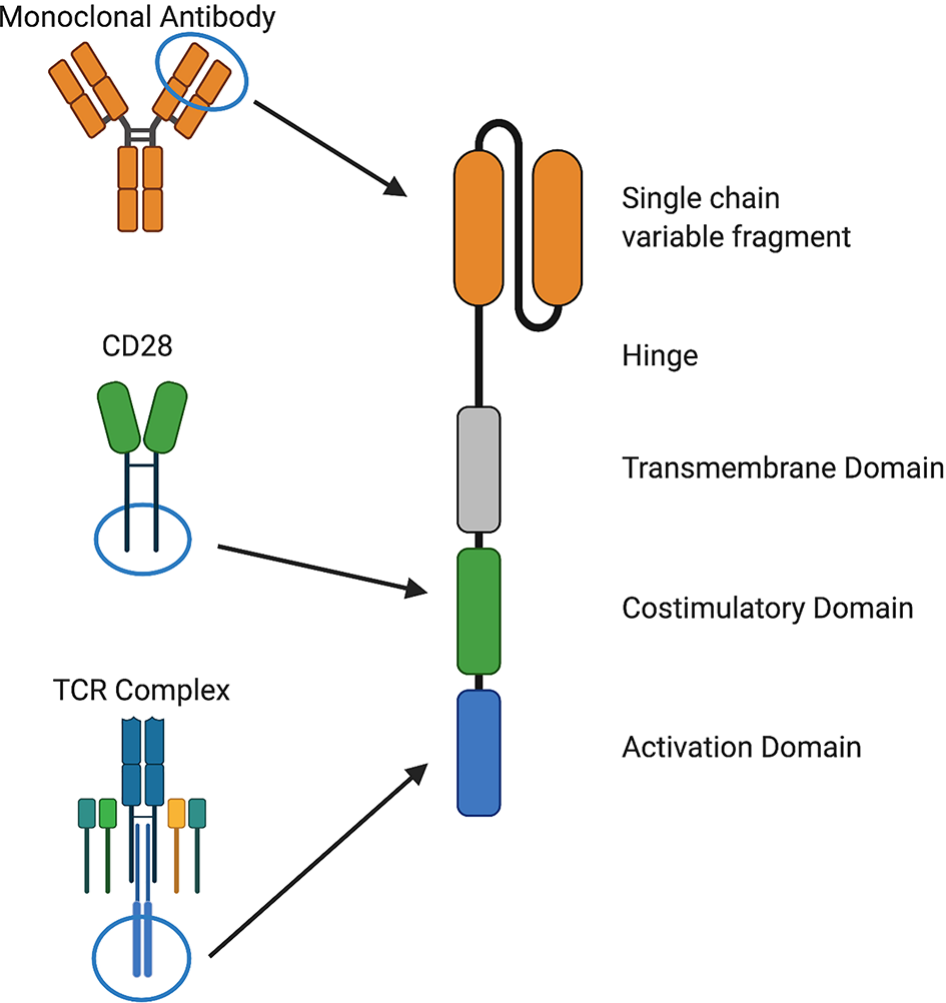
Components of chimeric antigen receptors. CARs recognize a cell surface antigen *via* a single chain variable fragment (scFv) from a monoclonal antibody or a ligand and signal through costimulatory domains derived from and CD28, 4-1BB, or other molecules and an activation domain derived from CD3ζ. Structural components, such as hinge and transmembrane domains derived from CD28, CD8α, or other molecules, are also important for CAR function.

Despite recent advances in cancer treatment, patients with relapsed or refractory disease continue to have poor outcomes and novel approaches are needed. T cells that are genetically modified to express a chimeric antigen receptor (CAR) can kill chemotherapy-resistant tumor cells and therefore have the potential to improve outcomes and reduce treatment-related toxicity from conventional therapies (1, 2). CARs consist of four components: i) an extracellular antigen recognition domain, most commonly a single chain variable fragment (scFv), ii) structural components, such as hinge and transmembrane domains, iii) a costimulatory domain that provides signals to sustain CAR T cell effector functions, and iv) a CD3ζ activation domain (1–3) (**Figure 1**).

CAR T cells targeting CD19 have shown significant overall response rates against CD19-positive leukemia and lymphoma (4–6), leading to their FDA approval in 2017. In addition, CAR T cells targeting BCMA, CD30, CD22, or CD20 expressed on hematological malignancies have also shown significant activity in clinical studies (7–10). However, a subset of patients does not achieve remission or relapses with antigen-positive disease due to suboptimal expansion or persistence of CAR T cells (11). CAR T cells for the treatment of solid tumors are also actively being explored, but they have shown less impressive clinical results (12–16), most likely due to a multitude of factors that limit CAR T cell activity. Previous preclinical studies have demonstrated that improvements in CAR design, such as optimizing scFvs, modification of structural components, or modulation of CAR signaling can improve the antitumor activity of CAR T cells (17–21). Likewise, additional genetic modification may be required to endow CAR T cells with potent and sustained effector function and to overcome the immunosuppressive tumor microenvironment (TME) to produce lasting benefits for cancer patients (22).

**Figure 2:**
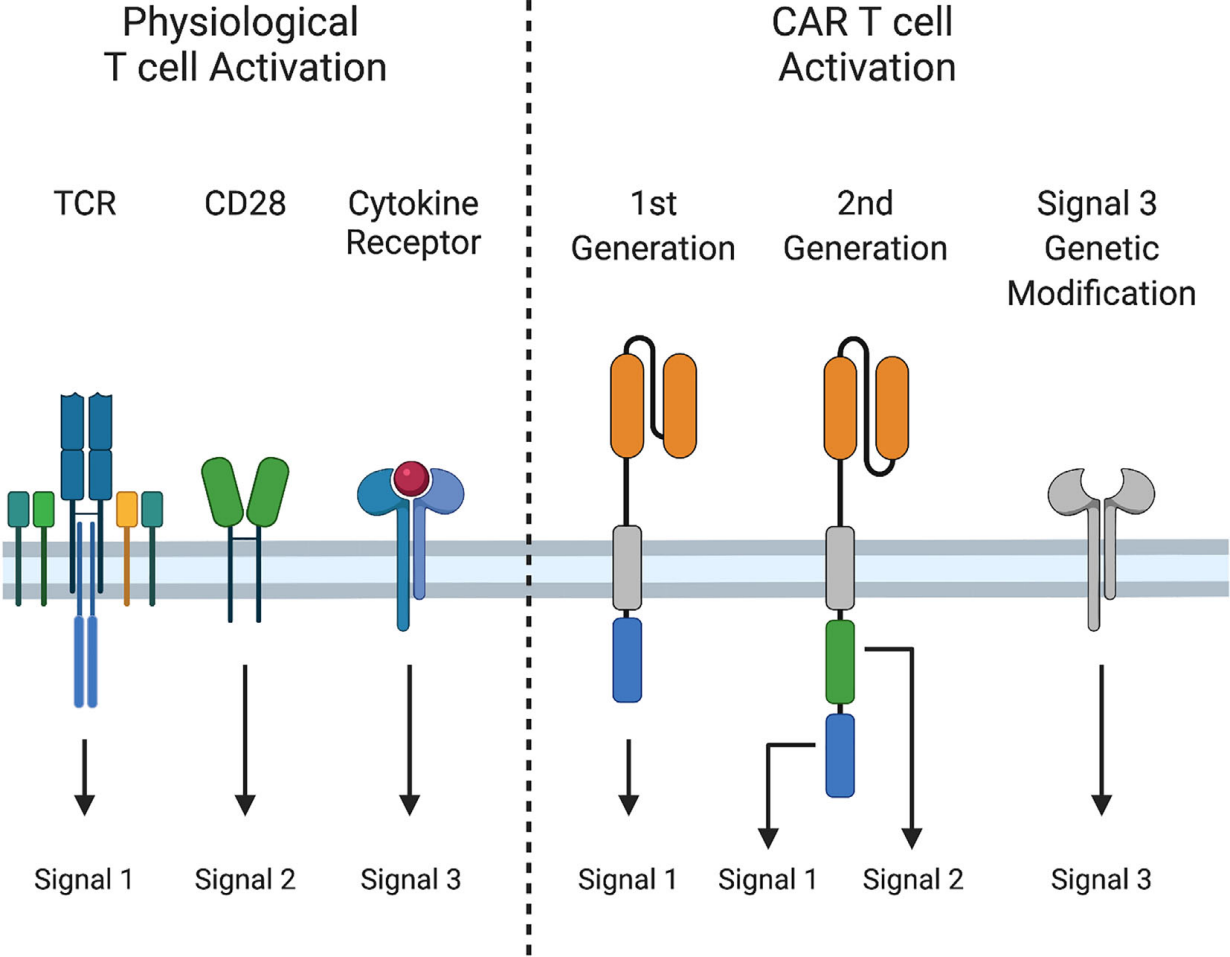
Signals 1, 2, and 3 in T cell activation. Physiological T cell activation and sustained effector function require i) Signal 1: peptide-MHC recognition and signaling through the T cell receptor (TCR), ii) Signal 2: costimulation through CD28, and iii) Signal 3: cytokine stimulation. First generation CARs provide only Signal 1, while second generation CARs provide Signals 1 and 2, and induce cytokine production (Signal 3). Signal 3 can be augmented by additional genetic modifications.

Physiological T cell activation requires three distinct signals for acquisition of effector function and formation of immunological memory. Signal 1 (activation) occurs *via* CD3ζ signal transduction following T cell receptor (TCR)-mediated antigen recognition. Signal 2 (costimulation) provides additional signals from CD28 or other molecules to augment signal 1. Finally, signal 3, mediated by cytokines, is required for optimal T cell proliferation, differentiation of naïve T cells into effector cells, and development of functional T cell memory (23–25) (**Figure 2**).

First generation CARs provide only signal 1, *via* CD3ζ. Second-generation CARs also provide signal 2, most often through CD28 or 4-1BB co-stimulation to sustain CAR T cell expansion following activation. Although activated CAR T cells produce cytokines, such as interleukin-2 (IL-2), production decreases after repeated exposure to tumor cells (26), and some cytokines that are important for T cell effector function, such as IL-12 and IL-15, are either produced at low levels are not at all by T cells (27, 28). Due to these limitations, investigators have engineered CAR T cells to augment signal 3. While incorporating cytokine receptor chains or JAK/STAT binding domains into CARs improves CAR T cell effector function (29, 30), most approaches to provide or modulate cytokine signaling have relied on transgenic expression of cytokines or cytokine receptors. In this review, we will provide a succinct overview of cytokine biology and highlight strategies to improve signal 3 in CAR T cells, including constitutive and inducible expression of cytokines and expression of native and engineered cytokine receptors.

## Common Gamma Chain Cytokines and Their Receptors

### Cytokine Biology

**Figure 3:**
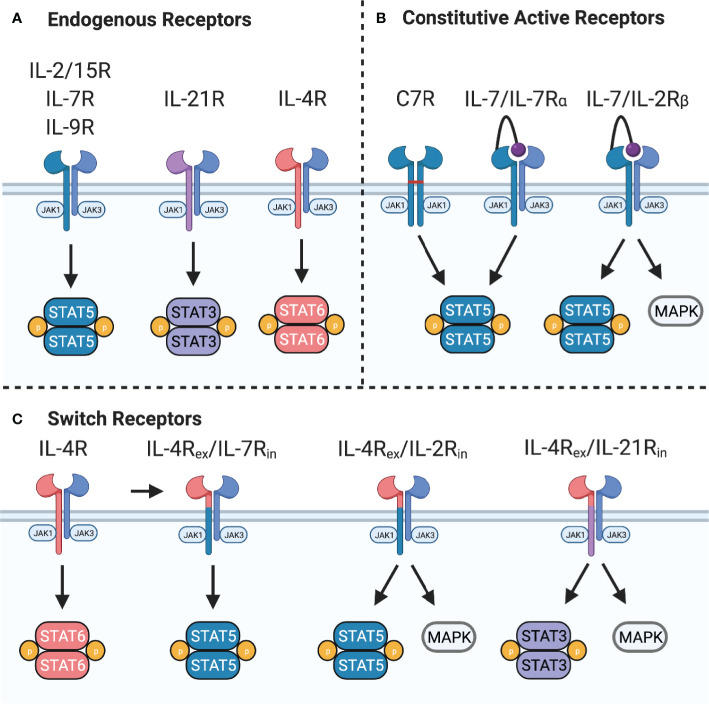
Common gamma chain cytokine signaling and synthetic receptors. **(A)** Common gamma chain cytokine receptors IL-2/15R, IL-7R, and IL-9R signal primarily through STAT5 while IL-21R signals primarily through STAT3. IL-4R signals through STAT6 to induce Th2 polarization. These receptors also activate PI3K and all except IL-7R activate MAPK pathways. **(B)** Constitutive active variants of IL-7R can provide constant STAT5 activation. C7R is formed from homodimerization of IL-7Rα chain mutants through a disulfide bridge in the transmembrane domain. A constitutively active IL-7R provides constant STAT5 and PI3K signaling by expression of IL-7 tethered to IL-7Rα. Switching the IL-7Rα intracellular domain with IL-2Rβ provides constant STAT5, PI3K, and MAPK signaling. **(C)** Switch receptors bind an immunosuppressive cytokine, such as IL-4, and convert it into a stimulatory signal. These receptors are composed of the IL-4Rα extracellular domain (IL-4R_ex_) fused to the IL-7Rα, IL-2Rβ, or IL-21R intracellular domain (IL-7R_in_, IL-2R_in_, or IL-21R_in_).

The common gamma chain family of cytokines – IL-2, IL-4, IL-7, IL-9, IL-15, and IL-21 – play critical roles in T cell differentiation, proliferation, and homeostasis. The receptors for these cytokines include the common gamma chain (γ_c_) and a private receptor chain (IL-4Rα, IL-7Rα, IL-9Rα, IL-21R) except for IL-2 and IL-15 that share γ_c_ and IL-2Rβ. Additionally, the IL-2/15 receptor can associate with IL-2Rα (CD25) or IL-15Rα to form high affinity IL-2 or IL-15 receptors. Binding of cognate cytokines induces heterodimerization of γ_c_ with the private receptor chain to position inactive Janus kinase (JAK)1 and JAK3 in proximity where they trans-phosphorylate each other to become active. Activated JAK1 and JAK3 subsequently phosphorylate the receptor to provide phosphotyrosine binding sites for SH2-domain containing proteins. Some of the primary signaling molecules activated downstream of these cytokine receptors are members of the Signal Transducer and Activator of Transcription (STAT) family (**Figure 3A**). In the case of the IL-2 receptor, phosphorylation of STAT5A and STAT5B by JAK1/3 induces homo- or hetero-dimerization and immediate nuclear translocation to induce expression of several cell cycle and anti-apoptosis genes, including Bcl-2, Bcl-x, Pim-1, c-myc, and cyclin D2 (31, 32). Additionally, mitogen-activated protein kinase (MAPK) and phosphatidylinositol 3-kinase (PI3K) pathways activated downstream of the IL-2R play roles in proliferation and metabolic regulation that may potentiate STAT5 signaling (33), but each receptor varies in its ability to activate these pathways.

An important aspect of γ_c_ receptor signaling is positive and negative regulation of the pathway to enhance or repress signaling circuits. For example, IL-2 upregulates expression of the high-affinity IL-2Rα chain, which can increase the sensitivity of T cells to IL-2, thus enhancing IL-2 signaling and T cell proliferation (34). Similarly, IL-4 induces expression of IL-4Rα, enforcing T helper (Th)2 polarization of CD4 T cells (35). Conversely, STAT5 signaling upregulates cytokine inducible SH2-containing protein (CISH) and suppressor of cytokine signaling (SOCS), which directly inhibit JAK activity and ubiquitinate the receptor complex, leading to proteasomal degradation (36).

While some of the γ_c_ cytokines can mediate similar signaling pathways and transcriptional programs (32, 37), physiological differences in cytokine signaling are mediated by competition for γ_c_ between the different receptors (38), variability in receptor expression on T cell subsets, a bias for signaling through different STAT molecules, and differences in activation of the MAPK and PI3K pathways (39, 40). The end result of γ_c_ cytokine receptor signaling is a transcriptional program that is influenced by activation of different STAT heterodimers, homodimers, and tetramers interacting with the chromatin landscape that differs in CD4 and CD8 T cells, as well as in naïve, memory, and exhausted T cells (41–43). Thus, future work to understand these transcriptional networks in CAR T cells could yield rational combinations to improve adoptive cell therapy for cancer.

### Interleukin 4 and 9

So far, IL-4 and IL-9 in the context of adoptive cell therapies have been understudied. IL-4 can have anti-cancer properties (44, 45), but it is largely immunosuppressive (46). IL-4 is involved in the differentiation of naïve CD4 T cells into IL-4-producing Th2 cells, which have been associated with less antitumor activity than IFNγ-producing CD4 T cells (Th1) (47). Adoptive transfer of tumor-specific IL-4-producing cytotoxic CD8 T cells (Tc2) was also less effective in controlling tumor growth compared to IFNγ-producing CD8 T cells (Tc1) (48–50). Additionally, several cancer types express IL-4 (51, 52) and the IL-4R (53–55), which suggests a role in tumor progression.

IL-9 was originally described as a T cell growth factor involved in the Th2 response, but recent studies have shown that IL-9 producing T cells (Th9/Tc9) can cause tissue inflammation (56) and inhibit tumor growth by activating mast cells (57) or by indirectly attracting immature dendritic cells (DCs) and activated CD8 T cells to tumors (58). Activation of the endogenous immune system by Th9 cells was better able to control tumor growth than conventional Th1 cells (58). Similarly, Tc9 cells were found to be superior to Tc1 cells for adoptive cell transfer by differentiating into effector cells that can persist longer *in vivo*, resist exhaustion, and resist apoptosis, which allows them to better control tumor growth (59). While there have been few publications evaluating the utility of IL-9 in improving CAR T cell therapy, Th9/Tc9-polarized human CAR T cells were recently shown to have superior antitumor activity compared to conventional Th1/Tc1 CAR T cells (60). These IL-9 producing T cells have several characteristics of an ideal population of T cells for adoptive cell transfer – they are hyperproliferative, cytotoxic, and resistant to exhaustion – which allows them to eradicate established tumors (61). However, there is conflicting evidence about whether IL-9 is dispensable (60–62) or indispensable (57–59, 62, 63) for antitumor responses. Further work will be required to evaluate this unique T cell population for adoptive cell therapies and to delineate if provision of IL-9 signaling is a general approach to improve CAR T cells.

### Interleukin 2 and 15

Early attempts to improve the antitumor activity of CAR T cells were based on the clinical use of IL-2 and IL-15 as anti-cancer immunotherapeutics, which as a monotherapy or in combination with adoptive transfer of tumor infiltrating lymphocytes (TILs) (64–66) had significant antitumor activity but were also associated with toxicity at higher doses (66–70). IL-2 and IL-15 promote immune cell proliferation and the transcription of anti-apoptotic proteins, but responsiveness to these two cytokines varies between Natural Killer (NK) cells and T cell subsets. The high-affinity IL-2 receptor is expressed by regulatory T cells (T_regs_) and activated CD4 and CD8 T cells, though memory CD8 T cells and NK cells are also responsive to IL-2 to a lesser degree. Conversely, memory CD8 T cells and NK cells are most responsive to IL-15, while T_regs_ and naïve CD4 and CD8 T cells are not. Constitutive expression of IL-2 by 1^st^ generation CD19-CAR T cells improved the tumor-free survival of mice with disseminated lymphoma compared to CAR T cells alone, although other γ_c_ cytokines were associated with greater improvements in survival and longer persistence of CAR T cells (71). However, the use of IL-2 has fallen out of favor due to the potential expansion of T_regs_ that can impede antitumor responses (72). Additionally, prolonged exposure to IL-2 promotes activation induced cell death (AICD) (73) and terminal differentiation of T cells into highly cytotoxic effector cells that can efficiently kill tumor cells, but are not able to sustain long-term antitumor activity *in vivo* that is associated with durable responses (74).

Constitutive expression of IL-15 improved the antitumor activity of CAR T cells specific for CD19, GPC-3, CLL-1, GD2 and IL-13Rα2 (75–79), likely due to a combination of greater expansion and persistence. Constitutive expression of IL-15 by first generation CD19-CAR T cells improved their *in vivo* antitumor activity and allowed cells to persist in mice for up to 110 days after tumor challenge (71). Notably, IL-15 expression maintained the greatest persistence of CD19-CAR T cells compared to other γ_c_ cytokines, and a subset of these persisting cells were memory-like T cells, a phenotype that is associated with sustained responses in patients treated with CAR T cells (80). The expansion or maintenance of memory T cell subsets, such as stem cell memory T cells (T_scm_), by IL-15 (81) could contribute to enhanced CAR T cell expansion and persistence. In addition to transgenic expression of secretory IL-15, expressing a membrane-bound form of IL-15 is an alternative way to provide pro-survival signals to CAR T cells (82). This approach is currently under evaluation in a phase 1 trial of CD19-CAR T cells (**Table 1**).

**Table 1:** Selected CAR T cell clinical trials with additional modifications to enhance signal 3.

Antigen	2^nd^ Genetic Modification	Cell Type	Indication	Clinical Trial ID
CD19	IL-7 + CCL19	T cells	B Cell Lymphoma	NCT03929107
CD19	IL-7 + CCL19*	T cells	Diffuse Large B Cell Lymphoma	NCT04381741
CD19	mbIL-15	T cells	B Cell Leukemia and Lymphoma	NCT03579888
EGFR	NFAT.IL-12	T cells	Colorectal Cancer	NCT03542799
ErbB	IL-4Rα/IL-2Rβ (4αβ)	T cells	Head and Neck Squamous Cell Carcinoma	NCT01818323
GD2	C7R	T cells	High Grade Glioma	NCT04099797
GD2	C7R	T cells	Solid Tumors	NCT03635632
GD2	IL-15	T cells	Neuroblastoma	NCT03721068
GD2	IL-15	NKT cells	Neuroblastoma	NCT03294954
GPC3	IL-7 + CCL19**	T cells	Hepatocellular Carcinoma	NCT03198546
Integrin β7, BCMA, CS1, CD38 or CD138	IL-7 + CCL19	T cells	Multiple Myeloma	NCT03778346
Nectin-4/FAP	IL-7 + CCL19 or IL-12	T cells	Solid Tumors	NCT03932565
MUC16ecto	IL-12	T cells	Ovarian Cancer	NCT02498912

*patients also receive PD-1 mAb (tislelizumab).

**subset of T cells are genetically modified with different transgenes.

No studies have compared head-to-head the efficacy of membrane-bound IL-15 to secreted IL-15 in engineered T cells, but evidence from preclinical studies suggests that membrane-bound IL-15 may be advantageous in certain situations. In addition to tumor-infiltrating CAR T cells, other immune cells, such as NK cells, NKT cells, endogenous T cells, and innate lymphoid cells (83), are responsive to IL-15. Secreted IL-15 could theoretically induce proliferation in these cell types but in CD8 T cells, membrane-bound IL-15 presented in the context of IL-15Rα, in contrast to secreted IL-15, provided sustained signal transduction that likely contributes to maintenance of memory CD8 T cells (84). Similarly, membrane-bound IL-15 tethered to CD8α improved the survival of engineered NK cells to a greater extent than expression of secreted IL-15, although this effect was due to autocrine signaling (85). Membrane-bound IL-15 tethered to IL-15Rα also improved survival and maintained a greater number of stem cell memory-like CAR T cells following withdrawal of antigen, compared to CAR T cells treated with IL-15/IL-15Rα complex (82). Nonetheless, many preclinical studies support transgenic expression of secreted IL-15 to improve CAR T cell antitumor activity. Ongoing clinical trials with IL-15 engineered T cells and further preclinical work could inform the optimal mode of IL-15 delivery to enhance adoptive cell therapy for cancer.

Concerns of uncontrolled proliferation or toxicity of CAR T cells expressing γ_c_ cytokines (86) have spurred the development of inducible safety switches in combination with T cell stimulating cytokines. Inducible caspase 9 (iC9) is an engineered protein that can be activated by a chemical inducer of dimerization to initiate apoptotic cell death (87), and activation of iC9 *in vivo* resulted in rapid depletion of genetically modified cells in humans (88). Several preclinical studies have shown that co-expression of iC9 and IL-15 improves CAR T cell function and allows the selective depletion of genetically modified CAR T cells. Based on these preclinical studies, GD2-CAR T cells co-expressing iC9 and IL-15 are in early phase clinical evaluation (**Table 1**). In addition to CAR T cells, CAR natural killer T (NKT) cells have been genetically modified to express IL-15 (89). CAR.IL15 NKT cells maintained a population of central memory-like cells that were less prone to exhaustion and apoptosis, which translated into improved antitumor activity *in vivo* (90). Additionally, IL-15 protected CAR NKT cells from inhibition in the hypoxic TME (91). Based on these studies, the safety and efficacy of GD2-CAR NKT cells co-expressing IL-15 is currently being evaluated in pediatric patients with neuroblastoma (**Table 1**), and an interim analysis of the first three patients found that infusion of CAR.IL15 NKT cells is safe and associated with clinical benefit (92). In addition, NK cells expressing CD19-CARs, iC9 and IL-15 have been successfully evaluated in one early phase clinical study (93).

### Interleukin 7

Subcutaneous administration of IL-7 induced dose-dependent increases in the number of circulating CD4 and CD8 T cells without causing serious adverse events, such as capillary leak syndrome, that were observed in patients treated with IL-2 or IL-15 (66, 69, 94). In addition to promoting homeostatic expansion of T cells, IL-7 has also been shown to increase TCR repertoire diversity by preferentially expanding naïve T cells and recent thymic emigrants, which could improve the formation of an anti-cancer immune response (95). Therefore, transgenic expression of IL-7 has been explored as an alternative method to improve CAR T cells without the limitations of IL-2. In fact, genetically modifying GD2-CAR virus-specific T cells to express IL-7 allowed them to kill target cells in the presence of inhibitory T_regs_, while these cells were suppressed in the presence of IL-2 (96). Constitutive expression of IL-7 improved the antitumor activity of CD19-CAR T cells compared to CAR T cells expressing IL-2 or IL-15, possibly as a result of sustained target cell killing and T cell expansion, however these cells did not exhibit the long-term persistence observed with IL-15-expressing CD19-CAR T cells (71). IL-7 similarly improved CD20- and mesothelin-CAR T cells, but this effect required co-expression of C-C Motif Chemokine Ligand 19 (CCL19) (97). Expression of both IL-7 and CCL19 increased intra-tumoral infiltration of CAR T cells, endogenous T cells, and DCs, which achieved complete regression of established solid tumors and led to the formation of a T cell memory response *via* epitope spreading. CAR T cells engineered to express IL-7 with CCL19 or CCL21 also had improved antitumor activity due to enhanced CAR T cell proliferation and chemotaxis (98). These studies have been translated into clinical trials to evaluate the safety and efficacy of IL-7 and CCL19 expressing CAR T cells against lymphoma, multiple myeloma, and solid tumors (**Table 1**).

The greatest limitation to this approach is the downregulation of IL-7Rα as a result of sustained IL-7 signaling or TCR stimulation (99). To restore responsiveness to IL-7, T cells can be modified to constitutively express IL-7Rα (100), but prolonged exposure to IL-7 in IL-7Rα transgenic T cells can promote apoptosis through IFNγ-induced upregulation of Fas and FasL (101). Alternative approaches have been explored to provide the benefits of IL-7 signaling including expressing constitutive active cytokine receptors (102).

### Interleukin 21

In contrast to other γ_c_ cytokines, IL-21 shows a preference for activation of STAT3 over STAT5 while also signaling through PI3K and MAPK pathways to mediate proliferation (39). Constitutive expression of IL-21 by 1^st^ generation CD19-CAR T cells improved overall survival of mice with disseminated lymphoma (71). The greatest improvement in overall survival was seen in CAR T cells expressing IL-21, compared to CAR T cells expressing IL-2, IL-7, or IL-15, even though these cells expressed lower levels of Bcl-2, IFNγ, and TNFα. The improved antitumor activity could be due to maintenance of less differentiated effector memory T cell (T_EM_) subsets and/or by enhancing long-term persistence. In accordance with this observation, IL-21 has been shown to decrease terminal effector differentiation of CD8 T cells, as judged by expression of granzyme B and EOMES, in comparison to IL-2 treated CD8 T cells, which resulted in improved *in vivo* antitumor activity (103). IL-21 also improved the expansion and persistence of adoptively transferred CD8 T cells resulting in superior antitumor activity in syngeneic tumor models compared to IL-2 and IL-15 (104). While the efficacy of IL-21-driven STAT3 signaling in T cell immunotherapy is still being explored, STAT3 signaling has been correlated with improved CD19 CAR T cell treatment outcomes in chronic lymphocytic leukemia (80).

### Constitutive Active Cytokine Receptors

An alternative strategy to enhance signal 3 of T cell activation is by transgenic expression of synthetic cytokine receptors (**Figure 3B**). For example, expression of a constitutively active IL-7 receptor (C7R) can overcome the downregulation of IL-7Rα due to negative feedback mechanisms and provide constant STAT5 signaling without a requirement for IL-7. Enhanced STAT5 signaling mediated by C7R was found to improve the *in vitro* and *in vivo* antitumor activity of GD2- and EphA2-CAR T cells (102) in an antigen-dependent fashion. C7R also increased tumor infiltration, expansion, and cytokine production of AXL-CAR T cells, but did not improve antitumor activity compared to the CAR alone (105). Clinical trials evaluating GD2-CAR T cells expressing C7R are in progress (**Table 1**).

Expression of IL-7Rα tethered to IL-7 is another way to provide cell-intrinsic IL-7 signaling (106). Since this is a modular platform for providing signal 3, the IL-7Rα intracellular domain can be replaced with other signaling domains. For example, investigators have expressed a chimeric IL-7Rα/IL-2Rβ receptor and IL-7 in CAR T cells to provide signal 3 and demonstrated that these engineered CAR T cells are resistant to TGFβ inhibition (107).

### Chimeric Cytokine Receptors

Chimeric cytokine receptors or switch receptors, which convert one cytokine signal into another, are actively being explored to hijack immunosuppressive cytokines produced by tumor or tumor-associated cells to provide proliferative signals to CAR T cells (**Figure 3C**). An IL-4/IL-7 switch receptor, which binds IL-4 but activates IL-7 signaling pathways, allowed PSCA-CAR T cells to maintain their cytolytic and proliferative capabilities *in vitro* and improved *in vivo* antitumor activity (108). In an orthotopic breast cancer model, the IL-4/IL-7 switch receptor also improved the antitumor activity of 2^nd^ generation MUC1-CAR T cells (109). Importantly, these cells were able to respond to tumor rechallenge at a distal site by proliferating and eliminating tumor cells in an IL-4-dependent manner. This approach has also been used to improve antitumor activity in the TME by converting immunosuppressive signals, such as TGFβ and IL-4, into separate stimulatory signals that improve tumor selectivity and CAR T cell potency (110). Expressing an IL-4/IL-2 switch receptor in MUC1- or PSMA-CAR T cells improved their cytolytic activity and proliferative capacity through increased STAT5 and ERK phosphorylation in the presence of IL-4 (111). Similarly, the receptor enhanced the antitumor activity and persistence of αvβ6 integrin-CAR T cells in an IL-4-dependent manner (112). An ongoing phase 1 clinical trial is investigating the utility of the IL-4/IL-2 switch receptor (4αβ) in CAR T cells for head and neck squamous cell carcinoma (**Table 1**).

While IL-4/IL-2 switch receptors convert STAT6 into STAT5 signals, IL-4/IL-21 switch receptors have been designed to convert STAT6 into STAT3 signals. This switch receptor improved cytolytic activity of GPC3-CAR T cells in the presence of IL-4, most likely by upregulating RORγt (113). Few studies have directly compared different cytokine switch receptors. However, comparison of IL-4/IL-7 and IL-4/IL-21 switch receptors in GPC3-CAR T cells demonstrated that in one solid tumor model, the IL-4/IL-21 switch receptor was superior (113). Lastly, TGFβ/IL-7 switch receptors have been expressed in PSMA-CAR T cells, improving their antitumor activity by upregulating AKT phosphorylation and Bcl-xL expression (114). In addition to tumor-derived cytokines that directly inhibit T cells, the TME can also produce cytokines for which T cells lack the corresponding receptor. For example, T cells lack the receptor for colony stimulating factor-1 (CSF-1). Engineering CAR T cells to express CSF-1R renders them responsive to CSF-1, which can promote chemotaxis, increase IFNγ production, and synergize with sub-optimal growth stimuli to enhance proliferation (115).

The orthologous IL-2 system is another way to modulate cytokine signaling in adoptively transferred cells. In order to limit the effects of the pleiotropic cytokine IL-2 to only select cell types, Sockolosky, *et al*. mutated IL-2 and IL-2Rβ (*ortho*IL-2 & *ortho*IL-2Rβ) such that the orthogonal cytokine/receptor pair can bind each other, but not the wild type IL-2 or IL-2Rβ (116). *In vivo, ortho*IL-2 could expand tumor-specific T cells expressing *ortho*IL-2Rβ to control tumor growth without the dose-limiting toxicity seen with high doses of IL-2. This approach could be adapted to drive other cytokine signaling pathways in engineered T cells without affecting endogenous immune cells.

## Interleukin 12 Family Cytokines and Their Receptors

### Cytokine Biology

**Figure 4:**
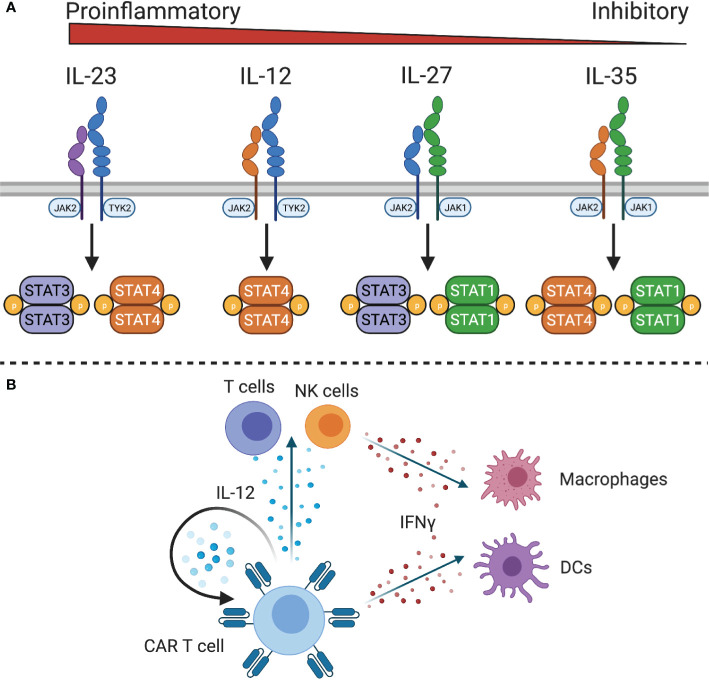
IL-12 family cytokine signaling. **(A)** The IL-12 family cytokine receptors signal through different STAT family members to exert proinflammatory or inhibitory functions. **(B)** IL-12 expressing CAR T cells can stimulate IL-12 responsive CAR T cells and endogenous T and NK cells. Downstream mediators of IL-12, such as IFNγ, can act on macrophages and dendritic cells to stimulate innate and adaptive immunity.

The IL-12 family of cytokines, IL-12, IL-23, IL-27, and IL-35, have diverse roles in innate and adaptive immune responses, with IL-12 and IL-23 being pro-inflammatory, IL-27 having both pro- and anti-inflammatory effects, and IL-35 being anti-inflammatory (117) (**Figure 4A**). Due to the primarily anti-inflammatory roles of IL-27 and IL-35, only IL-12 and IL-23 have been explored in the context of adoptive cell therapies.

Because of the role of IL-12 in linking adaptive and innate immunity, it has been explored as a potential cytokine to initiate or sustain antitumor immune responses. IL-12 is a heterodimeric protein composed of IL-12p40 and IL-12p35 that is produced by antigen presenting cells in response to microbial stimulation (118). The IL-12 receptor (IL-12R) consists of two chains, IL-12Rβ1 and IL-12Rβ2, which signals through JAK2 and tyrosine kinase (TYK)2 to activate STAT4 and other STAT family members to a lesser degree. It is primarily expressed by activated T cells and NK cells (119) although functional IL-12 receptor expression has also been observed on DCs (120), B cells (121), and myeloid-derived suppressor cells (MDSCs) (122). In both T cells and NK cells, IL-12 enhances proliferation, cytotoxicity, and the production of high levels of IFNγ, TNFα, and GM-CSF (123, 124). Additionally, IL-12 controls Th1 differentiation by directly inducing production of IFNγ and upregulating expression of IL-12Rβ2 *via* IFNγ-induced expression of T-bet, the Th1 defining transcription factor. This leads to a positive feedback loop wherein IFNγ enhances IL-12 production by DCs and renders T cells more sensitive to IL-12 to reinforce a Th1 phenotype. In addition to increasing IFNγ production, IL-12 also promotes expression of immunostimulatory cytokines and chemokines, such as GM-CSF, C-C motif ligands, IP10 (CXCL10), and MIG (CXCL9) to recruit T cells, NK cells, and antigen presenting cells to the site of inflammation (28, 125, 126). This has implications for cancer immunity by recruiting and activating effector cells, reprogramming tumor-associated macrophages to a pro-inflammatory phenotype (127), increasing antigen processing and presentation, reprogramming immunosuppressive MDSCs (128), and inhibiting angiogenesis (129).

IL-23 is a functionally related heterodimeric cytokine that is composed of IL-23p19 and the shared IL-12p40 subunit. The IL-23 receptor is composed of IL-12Rβ2 and IL-23R, which signals through JAK2 and TYK2 to primarily activate STAT3 and STAT4. Similar to IL-12, IL-23 is produced by antigen presenting cells and plays an important role in T cell differentiation. During T cell activation in the presence of cytokines such as TGFβ, IL-1, or IL-6, IL-23 upregulates expression of the IL-23 receptor and RORγt to reinforce a Th17 phenotype but is not required for Th17 differentiation. In the context of tumor immunity, IL-23 may play a distinct role compared to IL-12. The antitumor effects of IL-12 have been demonstrated (130), but IL-23 can have pro or antitumor properties, depending on the concentration of IL-23 and cancer type (131, 132).

### Interleukin 12

IL-12 has been used as a single agent immunotherapy and in combination with adoptive transfer of T cells to promote activation of tumor-specific T cells and differentiation into pro-inflammatory Th1/Tc1 cells. Systemic administration of IL-12 resulted in robust antitumor activity in preclinical models (133); however, clinical trials with recombinant human IL-12 were limited by modest efficacy and toxicity (134–137). Nonetheless, genetic modification of CAR T cells to secrete IL-12 is actively being explored. For example, modification of MUC16ecto-CAR T cells to constitutively secrete IL-12 enhanced IFNγ production and *in vivo* persistence, likely through an autocrine mechanism, leading to increased survival in an orthotopic xenograft model (138). Later work to define the interaction of tumor-associated immune cells and IL-12 producing CAR T cells confirmed that autocrine IL-12 signaling was necessary for antitumor activity by enhancing the effector function of CAR T cells, depleting tumor associated macrophages, and preventing tumor-mediated PD-L1 inhibition. Interestingly, IL-12 did not exert its effect in this model by recruiting endogenous T cells to the tumor or rely on host IFNγ producing cells (139). There are conflicting results about whether antitumor activity is primarily mediated by the effects of IL-12 on adoptively transferred T cells or host IL-12R expressing cells (128, 140). Nevertheless, a clinical study with MUC16ecto-CAR T cells expressing IL-12 is in progress (**Table 1**). Early results from this trial illustrate that IL-12 secreting MUC16ecto CAR T cells can be safely administered, although a high incidence of hemophagocytic lymphohistiocytosis/macrophage activation-like syndrome was observed when cells were administered after lymphodepleting chemotherapy (141).

The observation that constitutive expression of IL-12 by engineered T cells could promote T cell dysfunction (142–145) and toxicity in preclinical models (146) led to the design of vectors in which IL-12 expression is under the control of the Nuclear Factor of Activated T cell (NFAT) promoter (144). The NFAT promoter links IL-12 expression to T cell activation (144, 146), reducing systemic toxicity without decreasing antitumor activity (127, 146, 147). However, one clinical study with TILs modified to express NFAT-inducible IL-12 suggests that the NFAT promotor might not be sufficient to restrain IL-12 production to only activated TILs within the tumor (148). In addition to NFAT-controlled IL-12 expression, doxycycline-inducible IL-12 production is also actively being explored (149). While the autocrine effects of IL-12 contribute to improved antitumor activity of IL-12 CAR T cells, the importance of IL-12 in mustering an innate immune response cannot be overstated (**Figure 4B**). Endogenous T cells, NK cells, DCs, and MDSCs can respond to both IL-12 and the high levels of IFNγ produced by IL-12-responsive cells. One study found that inducible IL-12 allowed CAR T cells to eradicate antigen-positive tumor cells and prevent outgrowth of antigen negative tumor cells by recruiting and activating macrophages to produce TNFα and upregulate costimulatory molecules CD80/CD86 to enhance T cell responses (127). In another study, IL-12 was also shown to increase antigen processing and presentation (128), which could contribute to an endogenous antitumor immune response in solid tumors. Similarly, a study of IL-12 expressing VEGFR2-CAR T cells found that host IL-12R expressing cells, but not B or T cells, were required for the antitumor response (146). This activation of the innate immune system has the potential to produce lasting remissions for solid tumor patients, which is being evaluated in an ongoing phase I/II clinical trial of EGFR-specific CAR T cells with NFAT-inducible IL-12 to treat metastatic colorectal cancer (**Table 1**).

Additional approaches to recruit and activate innate immune cells to the TME are also under development. For example, CAR T cells engineered to secrete the DC growth factor Fms-like tyrosine kinase 3 ligand (Flt3L) were able to expand intra-tumoral conventional type 1 DCs (150). By engaging the endogenous immune system, Flt3L-CAR T cells, in conjunction with immune adjuvants, were able to mediate regression of established solid tumors and promote the formation of antitumor memory *via* epitope spreading. Approaches such as these will be necessary to overcome antigen heterogeneity and antigen-negative relapse in the treatment of solid tumors.

### Interleukin 23

While there have been few studies using IL-23 in combination with cell-based therapies, recent work has suggested that this is an option to improve CAR T cell function in solid tumors. CAR/TCR signaling upregulates expression of IL-23R and IL-23p19, but not the second subunit of IL-23 – IL-12p40. Transduction of T cells to constitutively express IL-12p40 leads to activation-induced expression of IL-23, but not IL-12, and improves T cell proliferation. CAR T cells engineered to express IL-12p40 exhibit greater expansion, persistence, and antitumor activity in xenograft and syngeneic tumor models (151), which could be attributed to an IL-23-induced STAT3 and hypoxia inducible factor (HIF) gene signature. STAT3 signaling in CAR T cell products has been associated with improved responses in chronic lymphocytic leukemia patients (80) and modulation of STAT3 signaling has been used to improve CAR T cells in other preclinical models (29).

## Interleukin 1 Superfamily of Cytokines

The IL-1 superfamily of cytokines – IL-1α, IL-1β, IL-33, IL-1 Receptor Antagonist, IL-18, IL-37, IL-36 Receptor Antagonist, IL-36α, IL-36β, IL-36γ, and IL-38 – plays important roles in innate and adaptive immunity, but only a subset of these cytokines has been evaluated in preclinical and clinical trials for their ability to elicit an antitumor immune response. For example, the role of IL-1β in cancer has been extensively studied, with different studies showing a role in either promoting or inhibiting tumorigenesis (152–155). Although IL-1β has the potential to improve adoptive cell therapy (156), IL-1β in conjunction with macrophage-derived nitric oxide and IL-6 plays a central role in cytokine release syndrome (CRS) (157, 158), a serious complication of CAR T cell therapy. However, other cytokines are emerging as anticancer mediators and their ability to improve CAR T cell therapy is currently under investigation.

### Interleukin 18

**Figure 5:**
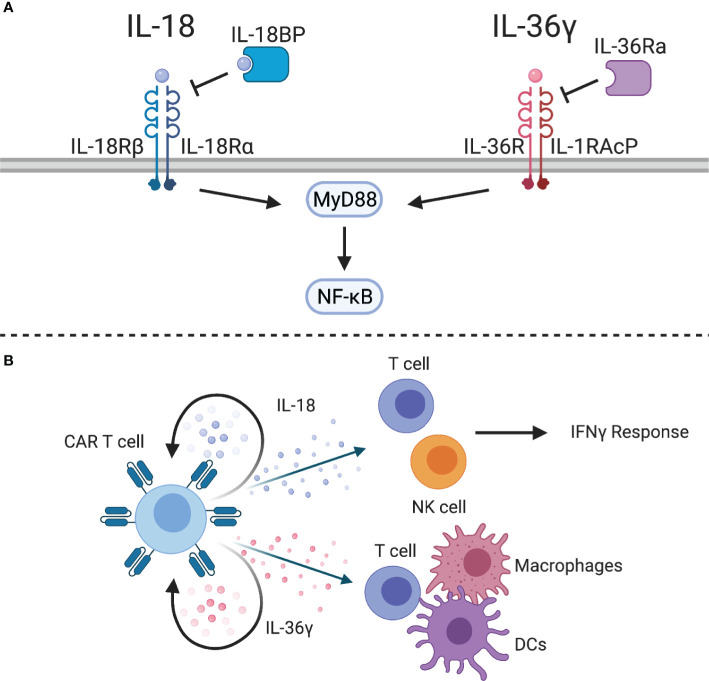
IL-1 superfamily cytokines. **(A)** The IL-1 superfamily cytokines, IL-18 and IL-36γ, signal through MyD88 to activate NF-kB. Inhibitory proteins, such as IL-18 binding protein (IL-18BP) or IL-36 receptor antagonist (IL-36Ra), can inhibit cytokine signaling. **(B)** IL-18-expressing CAR T cells can stimulate an IFNγ response from CAR T cells and endogenous T and NK cells. IL-36γ-expressing CAR T cells can provide autocrine signaling to enhance effector function and activate endogenous T cells, macrophages, and DCs.

IL-18 is a proinflammatory cytokine produced by macrophages, dendritic cells, epithelial cells, and other cell types that interacts with a heterodimeric receptor composed of IL-18Rα and IL-18Rβ expressed on NK cells and antigen-experienced T cells. IL-18 signals through MyD88 and NF-kB, and has been shown to have protumorigenic functions, such as promoting angiogenesis, metastasis, and proliferation (159), but it is thought to largely have antitumor activity due to its cooperation in the Th1 response (160). In conjunction with IL-12 or antigen stimulation, IL-18 induces the production of IFNγ and cytotoxic effector molecules by Th1 and CD8 T cells (**Figure 5A**) (161), resulting in activation of NK cells, macrophages, and other cell types. However, IL-18 signaling without concomitant inflammatory cytokines enhances Th2 responses, such as production of IL-4 and IL-13 (162, 163). Nonetheless, IL-18 has been tested in clinical trials for antitumor activity. While administration of IL-18 has been well tolerated (164), it showed no benefit in metastatic melanoma patients when given as a monotherapy (165).

Engineering human and murine CAR T cells to express IL-18 improved CAR/TCR-mediated proliferation, production of cytokines, and antitumor activity (166–169). One study highlighted the role of IL-18-secreting CD4 CAR T cells to promote expansion of CD8 CAR T cells (166). Another study highlighted that IL-18 induces a potent T cell effector subset characterized by a T-Bet^high^ FoxO1^low^ phenotype (167). Similar to IL-12, NFAT-inducible IL-18 expression systems have also been explored to limit systemic side effects associated with constitutive IL-18 expression (167, 168). In syngeneic models, IL-18 was able to remodel the TME with increased numbers of CD8 T cells, NK cells, and activated antigen presenting cells resulting in induction of tumor-specific T cell responses *via* epitope spreading (169). This was mirrored by a reduced number of immunosuppressive dendritic cells, M2 macrophages, and Tregs (167, 169). A possible limitation of transgenic IL-18 expression is the recent discovery of an IL-18 binding protein (IL-18BP), an immune checkpoint, that inhibits IL-18 signaling; however, approaches to develop IL-18BP-resistant IL-18 are actively being explored (170). Lastly, a recent study has highlighted that it is feasible to design chimeric switch receptors that activate IL-18 signaling pathways in CAR T cells (171).

### Interleukin 36

IL-36α, IL-36β, and IL-36γ are newly discovered members of the IL-1 superfamily that have shown potent antitumor activity in preclinical models (172, 173). These cytokines share a heterodimeric receptor, composed of IL-36R and IL-1RAcP, that signals through MyD88 and NF-kB (**Figure 5A**). Similar to IL-18, IL-36 cytokines can be inhibited by IL-36 receptor antagonist (IL-36Ra). While these cytokines have so far been understudied, they have the potential to improve adoptive cell therapy. For example, IL-36γ has been shown to transform the tumor microenvironment and mediate tumor-specific CD8 T cell responses (172). IL-36γ is produced by keratinocytes, epithelial cells, and immune cells, and exerts its functions on several cell types, including epithelial cells, macrophages, dendritic cells, and T cells (174). Engineering CAR T cells to express IL-36γ improves expansion and persistence, which results in improved antitumor activity compared to unmodified CAR T cells (175). While this effect was dependent on autocrine IL-36 signaling through MyD88 for initial tumor clearance, IL-36R is also abundantly expressed on myeloid cells. IL-36γ-expressing CAR T cells were able to enhance MHC class II and CD86 expression on splenic macrophages and DCs of tumor-bearing mice, which suggests that IL-36 plays a role in maturation of antigen presenting cells. Importantly, this induced antigen spreading as evidenced by tumor recognition by endogenous CD8 T cells. Therefore, IL-36γ-expressing CAR T cells could be a viable treatment option for solid tumor patients who often suffer from relapse related to antigen heterogeneity or antigen loss (**Figure 5B**).

## Discussion

Transgenic expression of individual cytokines and/or cytokine receptors has improved the effector function of CAR T cells in preclinical models. Studies have highlighted that transgenic cytokines do not only enhance the antitumor activity of CAR T cells but also modulate other cells within the TME and are able to induce or enhance endogenous tumor-specific immune responses. However, safety concerns have also been raised. CRS is a potentially fatal complication of CAR T cell therapy and secondary genetic modifications that enhance CAR T cell function could exacerbate CRS. A major concern with γ_c_ cytokines (IL-2, IL-7, IL-15, and IL-21) is enhanced proliferation of infused CAR T cells leading to production of toxic levels of effector molecules IFNγ and TNFα. Since CAR T cell peak expansion correlates with CRS (176), approaches to improve CAR T cell expansion must be weighed against the risk of dose limiting toxicities. While IL-6 and IL-1 play central roles in CAR T cell-induced CRS, high levels of IFNγ are also observed (177), and any cytokine that enhances IFNγ production by CAR T cells or other immune cells could potentially increase the incidence and/or severity of CRS. IL-12 can directly augment IFNγ production by T cells and NK cells. Similarly, IL-18 can synergize with IL-12 or IL-15 to enhance IFNγ production. IL-23 and IL-36 have not been implicated in CRS, but they play central roles in inflammatory conditions in the gut and skin and could therefore potentially contribute to CRS if produced at high levels by adoptively transferred immune cells.

While early phase clinical testing of CAR T cells secreting cytokines or constitutively active cytokine receptors are in progress, there are opportunities to further enhance this approach. A physiological immune response requires coordination of different cytokines to i) initiate an inflammatory reaction, ii) amplify responses of multiple cell types, and iii) resolve inflammation. However, the majority of studies have so far only explored constitutive expression of a single cytokine. Thus, developing approaches that endow CAR T cells with the ability to express an array of cytokines at different stages of CAR T cell activation holds the promise to not only increase efficacy but also safety. As these approaches are being developed, not only xenograft but also immune competent models are needed to careful analyze how cytokine-secreting CAR T cells engage with endogenous immune cells. Based on the breath of preclinical data generated thus far, we are confident that ‘signal 3-enhanced’ CAR T cells have the potential to improve the currently limited antitumor activity of CAR T cells in early phase clinical studies for patients with solid tumors and brain tumors.

## Author Contributions

MB and SG wrote, reviewed, and edited the manuscript. All authors contributed to the article and approved the submitted version.

## Funding

The work was supported by a grant from the National Cancer Institute 1F31CA250401-01A1, by a National Institutes of Health (NIH) grant R01NS106379 and the American Lebanese Syrian Associated Charities. The content is solely the responsibility of the authors and does not necessarily represent the official views of the NIH.

## Conflict of Interest

SG has patent applications in the field of T-cell and gene-modified T-cell therapy for cancer. He is a consultant for Catamaran Bio, Nektar Therapeutics, and TESSA Therapeutics, on the Scientific Advisory Board of Tidal, and a DSMB member of Immatics.

The remaining author declares that the research was conducted in the absence of any commercial or financial relationships that could be construed as a potential conflict of interest.
